# Individual work-motive values: determinants and consequences for the appraisal of specific health-related work characteristics

**DOI:** 10.3389/fpsyg.2024.1332687

**Published:** 2024-08-07

**Authors:** Stein Knardahl, Jan Olav Christensen

**Affiliations:** National Institute of Occupational Health, Oslo, Norway

**Keywords:** work-motive values, skill level, managerial role, work characteristics, self-leadership

## Abstract

The objectives of the present study were to determine whether (I) work-motive values influence the appraisal of specific work characteristics of significance for health and function and (II) subject variables impact work-motive values. Two aspects of work-motive values were studied: values that assign importance to pursuing one’s personal goals and interests, *internally based work-motive values* (IntWMVs), and values that assign importance to external factors, *externally based work-motive values* (ExtWMVs). These aspects of motive values, age, gender, skill level, managerial role, and specific psychosocial work characteristics were analyzed in a cross-sectional sample of 12,994 employees in 101 private and public organizations. Two-year follow-up prospective data from 6,252 employees in 69 organizations elucidated whether associations were stable over time. The results showed that IntWMV influenced reports of levels of control of decisions, empowering leadership, innovative climate, quantitative demands, feedback from work, and self-leadership. ExtWMVs were most consistently associated with role clarity. Skill level and managerial role were associated with reporting higher levels of IntWMVs and lower ExtWMVs. In conclusion, the present data support the assumption that work-motive values influence the appraisal, reporting, and consequently measurements of work characteristics. Managers differ from subordinates in work-motive values and may face challenges in ascertaining and supporting subordinates’ needs.

## 1 Introduction

Individuals differ in their styles of perception and appraisal; hence, individual-level factors influence the perception, appraisal, and reporting of work characteristics. Work-motive values is an individual-level factor that has received little attention, and there is a paucity of studies on its influence on the reporting of work characteristics. With reference to appraisal theory (Moors et al., 2013) and person-environment fit (P-E fit; Edwards and Cable, 2009; Kristof-Brown and Guay, 2011), the present study aimed to determine whether (1) work-motive values differentially influence the reporting of specific work characteristics that are known to impact health and function and whether (2) the subject variables management position, skill level, gender, and age affect work-motive values. The present study adds to the knowledge base of organizational and occupational psychology by elucidating the role of individual motive values in employees’ appraisal of their jobs. This knowledge is of methodological significance for the assessment of work characteristics and of potentially practical impact for understanding sources of variance and differences between managers and followers.

### 1.1 Values and motives

Value is an “enduring belief that… specific mode of conduct or end-state of existence is personally or socially preferable…” (Rokeach, 1973, p. 5). Beliefs pertaining to conduct were labeled *instrumental values,* while those pertaining to the end state of existence were labeled *terminal values*. Theories of personal values that aim to account for values in general organize values differently. Schwartz (1992) described two dimensions: (i) openness to change–conservation and (ii) self-enhancement–self-transcendence. Based on Schwartz’s theory, Arieli et al. (2020) provided a review of the impacts of personal values on behaviors, satisfaction, engagement, and wellbeing in organizations. Discussing the personal-value construct, they state that values differ from motives in that some motives are undesirable and that values represent conscious representations while people may be unaware of their motives (McClelland, 1985; Arieli et al., 2020). Work motives may be defined as individual preferences for outcomes from work (e.g., Sagie et al., 1996; Kooji et al., 2011). Clearly, these constructs overlap and we use the term ‘motive values’ to inform that the present study pertains to work motives that are reported by survey questions, that is, available to consciousness.

### 1.2 Motivation

Terminal values and motives relate to motivation and the constructs are sometimes confused. The American Psychological Association’s “APA Dictionary of Psychology” defines ‘motivation’ as “the impetus that gives purpose or direction to human or animal behavior and operates at a conscious or unconscious level” (VandenBos, 2007). Motivation is an abstract concept used to explain directional and activational aspects of behavior. The directional aspect of motivation contributes to processes of choice and initiation of behaviors, while the activational aspect determines the invested effort and intensity of behaviors toward attaining a goal. “Work motivation” is a general concept that encompasses both needs, motives, values, general attitudes toward one’s job, and specific motivation for specific work tasks or aspects of work (i.e., motivational state).

Theories of motivation are based on assumptions of fundamental drives, needs, desires, motives, values, or central nervous system functions (e.g., optimal arousal, Hebb, 1955; reduction of prediction error, Kaplan and Oudeyer, 2007). Hence, theories differ in assumptions of which fundamental factors drive motives, values, and motivational states. Maslow’s (1943) “Theory of human motivation” posits that humans are motivated by basic needs in a five-stage structure of priorities: (i) physiological needs, (ii) need for safety and predictability, (iii) need for love, (iv) need for self-esteem, and (v) need for self-actualization. McClelland’s “Need theory,” which is commonly applied by I/O-psychology consultants, proposes that people are primarily motivated by (i) the need for achievement, (ii) the need for power, or (iii) the need for affiliation to varying degrees (McClelland, 1961).

### 1.3 Extrinsic and intrinsic motivation

Several lines of experiments and observations confirm that both directional and activational aspects of motivation may be modified by external reinforcers. Motivation caused by extrinsic incentives and consequences of doing the task, that is, by a reinforcer, is commonly termed *extrinsic* motivation. External incentives are associated with work performance, particularly for quantitative-type tasks (Jenkins et al., 1998; Cerasoli et al., 2014). Transactional leadership theories are based on the motivational effects of social exchange, equity, and rewards (i.e., reinforcements).

Humans often immerse themselves in activities that are not reinforced by an external reward: The subject is motivated by the task *per se* and working on the task constitutes the motivation. *Intrinsic* motivation is inferred from engagement in a task for the inherent pleasure and satisfaction derived from the task itself. Pointing out shortcomings of drive-reduction theories, White (1959) proposed the concept of “competence,” defined as “… an organism’s capacity to interact effectively with its environment” (p 297) and “competence motivation” as an “intrinsic need to deal with the environment” (p 318). These concepts seem related to Maslow’s need for self-esteem and need for self-actualization and intrinsic motivation.

Self-determination theory (SDT, e.g., Deci et al., 2017) maintains that motivation is related to three innate psychological needs: (i) need for autonomy, (ii) need for competence, and (iii) need for relatedness. According to SDT, intrinsic motivation is the prototype impetus for self-determined behavior.

The role of reward and reinforcement is a pivotal issue for differentiating intrinsic from extrinsic motivation. The nature of reinforcement mechanisms may be elusive, however. Any sensory input signaling agency, novelty, or pleasure may act as reinforcers. For a baby watching his or her fingers move, both the visual and proprioceptive sensory input signals novelty and agency, which may be rewarding and hence reinforce play with fingers and hands. Play, hobbies, and work that are intrinsically motivating usually imply that the subject experiences tangible results of the effort and these outcomes can be intensely rewarding for her or him although other persons or the external surroundings do not ascribe any value to them. Furthermore, some seemingly intrinsically motivated activities may be escape from challenges or distressing environments (e.g., meditation activities and hobbies). A review by Morris et al. (2022) of “intrinsic vs. extrinsic motivation” presents an overview of models and discusses the operationalization of ‘intrinsic motivation’.

Several studies have concluded that *intrinsic* task motivation is associated with performance, particularly for qualitative-type tasks (Cerasoli et al., 2014; Deci et al., 2017). Transformational theories of leadership (Bass, 1985) assume that leaders can inspire and strengthen intrinsic motivation, possibly by internalization of goals and objectives.

### 1.4 Work motivation and motive values

Motivation plays a major role in translating human capital into productivity, and there is a plethora of theories and studies of factors that determine workplace motivational state and behaviors. Few of these theories discuss motives or values. One exception is the job characteristic model of motivation (JCM, Hackman and Oldham, 1976) that proposes that specific job characteristics such as task variation, task identity, task importance (representing meaning of work), control of one’s work situation (representing autonomy), and feedback (representing knowledge of results of one’s work) determine motivation and job satisfaction. This model recognizes that a “high need for personal growth and development” (labeled “growth need strength,” p 258) moderates relationships between job characteristics (task variation, identity, importance; autonomy; feedback) and outcomes like satisfaction in high-skilled workers (Hackman and Oldham, 1976; Loher et al., 1985). However, studies based on the JCM have not determined the direct effects of “growth need strength” on the reporting of work factors since the model treats job characteristics as independent variables (Hackman and Oldham, 1976; Loher et al., 1985).

Even if definitions vary between theories, it seems reasonable to assume that *intrinsic* task motivation depends on some degree of alignment between individual values or interests and the nature of the task at hand (cf. P-E fit principles). The goals or interests of an individual are values or motives primarily determined by cultural norms, values, education, previous experience, and personality (Salmela-Aro et al., 2012; Atherton et al., 2021). The general value of assigning importance to pursuing one’s personal goals and interests at work—where one’s motivation and satisfaction are generated by pursuing one’s personal goals and interests—seems related to the notion of motives for personal development (competence, White, 1959), personal ethical standards, need for self-actualization (Maslow, 1943), and “growth need strength” (Hackman and Oldham, 1976; Loher et al., 1985). For the purpose of the present study, we label this value *internally based work-motive values* (IntWMVs). Presumably, individuals with high levels of IntWMVs seek work that provides possibilities for intrinsic task motivation. The values that assign importance to safety, security, and income, that is, that motivation and satisfaction are generated by external factors, are labeled *externally based work-motive values* (ExtWMVs).

### 1.5 Perception and appraisal as method factors

Perception and appraisal are fundamental factors in interpreting and responding to one’s environment (e.g., Lazarus, 1991). The individual cognitive style of perception and appraisal may possibly be influenced by values. The appraisal of tasks and events is a pivotal determinant of people’s sentiments, opinions, and attitudes toward their work. Processes of perception and appraisal play roles in causal pathways from exposures to outcomes since psychological and biological responses depend on what is perceived and how this information is processed. This perspective maintains the significance of subjective appraisal for behavior and health.

On the other hand, one may argue that objective knowledge of exposures and work characteristics is important for risk assessment and measures to improve organizations. Perception and appraisal processes may result in inaccurate information about the objective reality. A substantial portion of the knowledge base of psychology is based on reports by individuals of their perception and appraisal of the phenomena under study, that is, subjective reports (Bodner, 2006) and surveys are the most prevalent data collection methods used by organizations (Rivers et al., 2009). For both science and practical applications of assessment of work (or other life exposures), the role of appraisal processes for potential information bias is a major methodological issue. Method factors that influence the subject’s response introduce method variance and/or bias of estimates of the construct that is measured (method bias; see Podsakoff et al., 2012). One established method factor is the personality characteristics of the individual. Neuroticism predisposes individuals to report mental and somatic symptoms (e.g., Cuijpers et al., 2010; Vassend et al., 2018) and influences the appraisal of social support (Swickert and Owens, 2010). Social desirability (self-deception; e.g., Nederhof, 1985) may influence perception and appraisal processes. Response styles (Knowles and Condon, 1999; Baumgartner and Steenkamp, 2001), instrument-design effects (Krosnick and Presser, 2010), and context factors at the time of responding that influence affective state (Askim and Knardahl, 2021) may influence responding to survey instruments.

Some appraisal theories take “motivational relevance” (Lazarus, 1991) or “consistency with motives” (Roseman et al., 1996) into account as one of the factors in appraisal processes. Perceiving discrepancies between what one experiences at the workplace and one’s values can be a source of discontent or distress (Arieli et al., 2020). George and Jones (1996) reported that a sum score of value attainment, job satisfaction, and positive mood interacts to determine turnover intentions. It seems plausible that motive values may play a fundamental role in the appraisal of work characteristics. Studies, primarily from one research group, have reported generally favorable effects of “predominant intrinsic work value orientations” on work-related outcomes such as job satisfaction and emotional exhaustion (Vansteenkiste et al., 2007). Surprisingly, we have not found studies of the effects of motive values on the appraisal and reporting of specific work characteristics.

### 1.6 Work characteristics

Occupational health studies of work factors that contribute to employees’ health, wellbeing, work ability, and absenteeism have consistently reported effects of the broad dimensions of job demands and control (Karasek, 1979; Kivimäki et al., 2012; Knardahl et al., 2017), “effort and reward” (Siegrist, 1996), and more specific factors such as social support from superior, role conflict, and fair leadership (e.g., Christensen and Knardahl, 2010; Elovainio et al., 2013; Finne et al., 2016). Studies generally take age, gender, and education into account in their analyses. Based on appraisal theories’ concepts of “motivational relevance” (Lazarus, 1991) or “consistency with motives” (Roseman et al., 1996), hypothetically, individual work-motive values may profoundly influence one’s perception, appraisal, and reporting of work characteristics, constituting both a theoretical and methodological challenge to studies.

### 1.7 Aims and objectives

The overarching aim of the present study was to determine whether work-motive values influence the perception and appraisal of some of the work characteristics that contribute to the wellbeing, function, and health of employees. We investigated an array of characteristics encompassing both psychological task-related factors (work content), social-interaction factors (with leader and co-workers), and self-leadership for the following reasons: (a) hypothetically, motive values may show distinct relations to specific work factors, (b) practitioners need information pertaining to specific and malleable factors to detect challenges and design measures for improvement, and (c) including the broader spectrum of factors in the same study enables determining which associations are robust and of practical significance. Finally, by selecting one or a very small number of variables from a larger set, one runs the risk of reporting statistically significant effects of limited practical impact (“cherry picking”). We assessed the effects of internally and externally based motive values and total motive intensity.

Work values may change with seniority and age, and work-motive values may contribute to aging employees’ motivation to stay or exit from work (Kooji et al., 2011). Therefore, one aim of the present study was to determine the effects of age, managerial position, skill level, and gender on work-motive values.

Internally based work-motive values (IntWMVs) pertain to seeking personal development, attaining personal goals and interests, and adhering to personal ethical standards. It seems reasonable to expect that perceived barriers or facilitators of personal development may be particularly relevant for individuals who prioritize this value. Specifically, one objective of the present study was to determine whether IntWMVs influence the perception and appraisal of levels of control of decisions at work, empowering leadership, and innovative climate. Having decision latitude or autonomy (control of one’s work situation) should be relevant for pursuing one’s personal goals and interests. Leaders (managers) differ in behaviors related to promoting employees’ development, participation in decisions, and autonomy, that is, empowering leadership. An innovative climate is the shared perception of conditions for innovativeness. Individuals motivated by personal development and attaining personal goals and interests may want to work in an innovative climate. The perception of rigid rules and conventions with little possibility of change may be seen as barriers to personal development. Therefore, IntWMVs may possibly influence the perception and appraisal of innovative climate.

Self-leadership refers to the employee’s autonomous behaviors (see Stewart et al., 2011, for review). Stronger IntWMVs may possibly motivate and promote autonomous behaviors resulting in more self-leadership. On the other hand, experiencing self-leadership may hypothetically influence work-motive values. Hence, we tested associations between the level of IntWMVs and self-leadership.

Since externally based motive values (ExtWMVs) may be related to instrumentally based motivation, one may assume that this value is related to input–outcome relationships (Adams, 1963). Self-reported job demands represent the employee’s appraisal of quantitative or qualitative requirements that he or she must fulfill in the job. Hence, job demands constitute a major aspect of the input dimension of input–outcome (equity) models (Adams, 1963).

To some degree, the evaluation of input–outcome relationships depends on receiving feedback or rewards for effort or performance, that is, the feedback that the employee receives at work. Hypothetically, having predominantly ExtWMVs may influence the subjective importance placed on receiving positive feedback and hence the perception and appraisal of feedback.

Externally based work-motive values are associated with needs and interests that are, in principle, unrelated to the work-task contents *per se*, such as status, security, and safety in addition to input–outcome relationships (Adams, 1963). Being treated fairly is important to most people, but one may hypothesize that having predominantly ExtWMVs may influence the perception of fair leadership. Furthermore, employees with ExtWMVs may emphasize the importance of safe and supportive social interactions at work. Specifically, we predicted that the level of ExtWMVs is associated with reported levels of job demands, feedback from work, levels of fair leadership, and social support.

Conflicts by nature imply sustained challenge (until resolved) and constitute health risks even if the subject seems to exhibit optimal behavioral coping responses (e.g., Lawler et al., 1980). Role conflict (i.e., conflicting expectations, standards, and demands) is a common type of conflict in working life that is associated with negative consequences for health (e.g., Christensen and Knardahl, 2010) and exit from working life (Emberland et al., 2017). Since role conflicts and ambiguity are significant predictors of health and wellbeing, we tested the hypothesis that levels of work-motive values are associated with reported levels of role expectations.

Individual response styles may influence responding. Therefore, we tested the hypothesis that the general level of activational motive values (i.e., total motive-value score = IntWMV + ExtWMV) represents a general motivational pattern that influences the perception and appraisal of work. We investigated both the strength of the two work-motive values (levels; IntWMVs and ExtWMVs) and the relative contribution of the IntWMV (fraction of total, f-IntWMV = IntWMV/total motive value score).

Since one primary objective was to determine the effects of (conscious) motive values on the perception and appraisal of work characteristics, we primarily based conclusions on responses given in the same survey, that is, on cross-sectional data. However, we also analyzed 2-year follow-up prospective data to elucidate whether associations were robust and stable over time.

## 2 Methods

### 2.1 Study design and population

The study was part of the project “The new workplace: work factors, sickness absence, and exit from working life” with a full-panel prospective design (all factors measured at all survey waves; Christensen and Knardahl, 2010; Nielsen et al., 2016). Organizations were recruited from 2004 to 2019; hence, the first measurement survey took place within this extended period. Private and public organizations participated (municipalities, government ministries, federal agencies, healthcare, finance, insurance, education, and non-profit organizations). All current employees of each organization were invited to participate (organizational-level convenience sampling). For those organizations that took part in two survey waves, the interval between waves ranged from 17 to 36 months (an average of 24 months, the second survey took place between 2006 and 2019). The surveys were primarily web-based (approximately 15% responded on a paper version). The information to participants contained no information on hypotheses or research questions.

The study was approved by the Norwegian Regional Committee for Medical and Health Research Ethics and the Norwegian Data Inspectorate and conducted in accordance with the Declaration of Helsinki.

Two samples were defined for the current analyses: a *cross-sectional* sample for which all employees in companies that participated at least once were eligible and a *prospective* sample comprising employees from companies that participated at least twice (Table 1). The *cross-sectional* sampling frame consisted of 26,841 invited employees of 1,482 work units in 101 companies. Of these, 14,679 individuals (54.7%) completed all items about motivational attitudes, and 10,971 (40.9% of all invited) also completed all items pertaining to at least one work factor as well as sex, age, skill level, and management position.

**Table 1:** Descriptive statistics: subject characteristics of cross-sectional analyses (*N* = 10,971).

	*N*	%
Sex		
Male	4,992	46
Female	5,979	54
Age		
< 30	1,196	11
30–50	6,681	61
> 50	3,094	28
Skill level		
< 10 years	107	1
10–12 years	3,850	35
13–15 years	2,748	25
> 15 years	3,075	28
Managers or unspecified	1,191	11
Managerial role		
No managerial role	8,705	79
Middle manager	1989	18
Top manager	277	3

The *prospective* sampling frame comprised 15,580 invited employees of 986 work units in 69 companies. Of these, 6,997 (44.9%) individuals provided information about motivational attitudes at both time points, and 5,437 (34.9%) also provided information about at least one work factor as well as skill level, age, sex, and management position.

### 2.2 Assessment of work-motive values

Work-motive values were measured with seven questions from The General Nordic Questionnaire for Psychological and Social Factors at Work (*QPSNordic*; Dallner et al., 2000): “How important are the following considerations in relation to your ideal job?”

Three items measured internally based work-motive values (IntWMVs): (1) to develop my own personality, (2) to get a sense of accomplishing something worthwhile, and (3) to be able to put my imagination and creativity to good use at work. To eliminate the possibility that high levels of IntWMVs were caused by the general strength of work-motive values or a general tendency for reporting higher levels (response styles), we also tested the effects of internal motive values as the fraction of total motive values.

Four items measured externally based work-motive values (ExtWMVs): (1) to have good pay and material benefits, (2) to have a peaceful and orderly job, (3) that the work is secure and provides regular income, and (4) to have a safe and healthy physical work environment. Response categories were (1) unimportant, (2) not so important, (3) rather important, (4) very important, and (5) absolutely necessary. Cronbach’s alpha for IntWMV was 0.66. Cronbach’s alpha for ExtWMV was 0.65. The original QPS_Nordic_ excluded the question “good pay and material benefits” from its external motive scale since it exhibited a moderate correlation with the scale (*r* = 0.34; Dallner et al., 2000). The values of Cronbach’s alpha were below a conventional cutoff of 0.7. However, the present measures consisted of relatively few items (alpha increases with a higher number of items). Moreover, the extent to which a high alpha is important, and how it should be interpreted, has been debated (Taber, 2018). Alpha may be seen as the extent to which the factor reflects a common, general construct, as opposed to the unique content of each item. Hence, when using few items to cover a relatively broad domain that comprises aspects that differ in meaning (e.g., good pay may differ from secure and safe), a lower alpha may be seen as acceptable and even expected.

To elucidate the potential effects of the general strength of motive values or a general tendency for reporting higher levels (response styles, Baumgartner and Steenkamp, 2001), we tested the effects of the sum of all seven motive-value items (total work-motive value score).

### 2.3 Reports of work characteristics

The QPSNordic has been extensively validated, has shown good psychometric properties (Dallner et al., 2000; Wännström et al., 2009), and provides a comprehensive assessment of key work factors. The following factors were assessed in the present study: *control of decisions* (5 items), *empowering leadership* (3 items), *innovative climate* (3 Items), *quantitative demands* (time pressure, amount of work; 4 items), learning *demands* (3 items), *feedback from work* (2 items), *fair leadership* (3 items), *social support from immediate superior* (3 items), *support from co-workers* (2 items), *role clarity* (3 items), *role conflict* (3 items). Cronbach’s *α* ranged from 0.71 for role conflict to 0.87 for empowering leadership. The two support-from-co-workers items exhibited Pearson’s *r* = 0.66.

Since most work factors may vary over time, response categories of the QPSNordic are frequency of occurrence (five levels, “very seldom or never”–“very often or always”) for all scales except *feedback from work* and *innovative climate* (five categories, “very little or not at all”–“very much”).

*Self-leadership* was measured with five items from studies by Houghton and Neck (2002) with five response categories: “very little or not at all”–“very much.”

### 2.4 Individual respondent characteristics

*Gender* and *age* were determined from Norwegian official social identity codes*. Skill levels* were determined based on occupations, according to a Norwegian adaptation of the International Standard Classification of Occupations (ISCO-88), by Statistics Norway. This classification expresses educational levels or equivalent levels of work experience typically required for different occupations (Christensen and Knardahl, 2010). Skill level also serves as a proxy for socioeconomic status. *Managerial role* was determined from one survey question. These factors were included as covariates in all analyses of associations between motivational attitudes and reported work factor levels.

### 2.5 Statistical analyses

*Subject variables*: In the random intercept linear regression models, subject characteristics were independent factors, and IntWMVs, ExtWMVs, and total motive-value scores were dependent factors, respectively. Prospective regressions were adjusted for baseline level of motive values.

*Work characteristics*: In the random intercept linear regression models, IntWMVs, internally based motive values as fraction of total motive-value score (f-IntWMV), and ExtWMV motive values, were independent factors, while work characteristics were dependent factors. All regressions were adjusted for skill levels, age, and gender. Prospective regressions are adjusted for baseline level of the respective outcome.

Due to the large number of analyses, we chose a *p*-value of <0.01 as the criterion for statistical significance (tables also present 95% confidence intervals). Recent years have seen criticism of basing conclusions solely on statistical analyses showing *p*-values lower than a standard criterion (Wasserstein et al., 2019). Therefore, we based conclusions on combined evaluations of *p*-values and estimates.

## 3 Results

### 3.1 Influence of demographic factors on work-motive values

Age was modestly associated with motive values. Female employees reported higher levels of both IntWMVs and particularly ExtWMVs, that is, they reported higher total motive value scores (Table 2). Skill levels >13 years were positively associated with IntWMVs and negatively associated with ExtMWV but only weakly associated with total motive scores (Table 2). Being a manager was associated with work-motive values: top managers exhibited a strong positive association with IntWMVs and a strong negative association with ExtWMVs (Table 2).

**Table 2:** Associations between age, gender, skill level, and manager roles with internally based work-motive values (IntWMVs), externally based work-motive values (ExtWMVs), and total work-motive value score (cross-sectional analyses).

Independent	IntWMV		ExtWMV		Total work-motive value score	
	Estimate	95% CI	Estimate	95% CI	Estimate	95% CI
Age	0.001	[0.000, 0.003]*	0.003	[0.002, 0.004]**	0.002	[0.002, 0.003]**
Female	0.089	[0.054, 0.113]**	0.188	[0.165, 0.210]**	0.148	[0.129, 0.166]**
Skill 10–12 years	Ref	–	Ref	–	Ref	–
Skill <10 years	−0.194	[−0.138, −0.070]**	0.093	[−0.018, 0.204]ns	−0.023	[−0.117, 0.072]ns
Skill 13–15 years	0.125	[0.090, 0.160]**	−0.041	[−0–072, −0.010] *	0.034	[0,008, 0.061]**
Skill >15 years	0.193	[0.158, 0.228]**	−0.126	[−0.158, −0.095]**	0.013	[−0.013, 0.039]ns
Managers and unspecified	0.137	[0.086, 0.188]**	−0.139	[−0.186, −0.092]**	−0.018	[−0.057, 0.021]ns
Not manager	Ref	–	Ref	–	Ref	–
Middle manager	0.124	[0.089, 0.159]**	−0.042	[−0.075, −0.010]**	0.029	[0.002, 0.057] *
Top manager	0.280	[0.197, 0.363]**	−0.243	[−0.320, −0.167]**	−0.020	[−0.085, 0.044]ns

IntWMVs, internally based work-motive values. ExtWMVs, externally based work-motive values. **p < 0.01 and *p < 0.05, ns: non-significant.

### 3.2 Effects of the strength of internally based work-motive values (IntWMVs) on the reporting of work characteristics

IntWMVs score strongly influenced reported levels of control of decisions, empowering leadership, and innovative climate, both cross-sectionally and prospectively (Tables 3, 4). Hence, our hypotheses regarding positive associations between IntWMVs and these specific work factors were supported. However, IntWMVs were also positively associated with quantitative demands, learning demands, feedback from work, role conflicts, fair leadership, social support from both superiors and co-workers, and role conflicts.

**Table 3:** Associations between work-motive values and reports of work characteristics (cross-sectional analyses).

Independent	IntWMV		f-IntWMV (fraction of total)		ExtWMV	
	Estimate	95% CI	Estimate	95% CI	Estimate	95% CI
Control decisions	0.201	[0.178, 0.223]**	0.925	[0.803, 1.046]**	−0.085	[−0.110, −0.060]**
Empowering leadership	0.162	[0.130, 0.194]**	0.610	[0.440, 0.779]**	−0.015	[−0.050, 0.019]ns
Innovative climate	0.132	[0.110, 0.155]**	0.253	[0.131, 0.375]**	0.075	[0.051, 0.100]**
Self-leadership	0.436	[0.413, 0.458]**	1.616	[1.493, 1.740]**	−0.027	[−0.051, −0.003] *
Quantitative demands	0.116	[0.093, 0.139]**	0.436	[0.315, 0.557]**	−0.016	[−0.041, 0.009]ns
Learning demands	0.100	[0.080, 0.121]**	0.346	[0.237, 0.455]**	0.005	[−0.017, 0.027]ns
Feedback from work	0.155	[0.126, 0.184]**	0.555	[0.401, 0.709]**	−0.004	[−0.035, 0.028]ns
Fair leadership	0.035	[0.018, 0.052]**	0.052	[−0.04, 0.144]ns	0.029	[0.010, 0.047]**
Social support from superior	0.058	[0.028, 0.088]**	−0.018	[−0.179, 0.144]ns	0.088	[0.055, 0.121]**
Social support from co-workers	0.075	[0.050, 0.100]**	0.035	[−0.098, 0.168]ns	0.083	[0.056, 0.110]**
Role clarity	−0.010	[−0.033, 0.013]ns	−0.507	[−0.631, −0.383]**	0.171	[0.146, 0.196]**
Role conflicts	0.064	[0.039, 0.089]**	0.379	[0.246, 0.512]**	−0.063	[−0.090, −0.036]**

IntWMVs: internally based work-motive values. ExtWMVs: externally based work-motive values. f-IntWMV (fraction of total): IntWMV/total work-motive value score.**p < 0.01 and *p < 0.05, ns: non-significant.

**Table 4:** Prospective analyses: associations between work-motive values and reports of work characteristics.

Independent	IntWMV		f-IntWMV (fraction of total)		ExtWMV	
	Estimate	95% CI	Estimate	95% CI	Estimate	95% CI
Control decisions	0.048	[0.022, 0.075]**	0.227	[0.086, 0.369]**	−0.020	[−0.049, −0.009]ns
Empowering leadership	0.058	[0.019, 0.097]**	0.319	[0.114, 0.525]**	−0.045	[−0.088, −0.003] *
Innovative climate	0.058	[0.030, 0.087]**	0.207	[0.058, 0.357]**	−0.009	[−0.040, 0.220]ns
Self-leadership	0.143	[0.114, 0.173]**	0.571	[0.419, 0.722]**	−0.040	[−0.071, −0.010]**
Quantitative demands	0.057	[0.030, 0.083]**	0.281	[0.143, 0.420]**	−0.035	[−0.064, −0.007] *
Learning demands	0.028	[0.003, 0.053] *	0.152	[0.020, 0.284] *	−0.019	[−0.046, 0.008]ns
Feedback from work	0.047	[0.012, 0.083]**	0.225	[0.038, 0.412] *	−0.028	[−0.066, 0.011]ns
Fair leadership	−0.001	[−0.023, 0.022]ns	−0.050	[−0.172, 0.072]ns	0.015	[−0.011, 0.040]ns
Social support from superior	0.011	[−0.026, 0.048]ns	0.013	[−0.183, 0.210]ns	0.005	[−0.036, 0.045]ns
Social support from co-workers	0.023	[−0.008, 0.053]ns	0.128	[−0.033, 0.288]ns	−0.018	[−0.051, 0.016]ns
Role clarity	−0.021	[−0.048, 0.006]ns	−0.293	[−0.438, −0.149]**	0.081	[0.051, 0.111]**
Role conflicts	0.026	[−0.004, 0.055]ns	0.119	[−0.040, 0.277]ns	−0.015	[−0.047, 0.018]ns

All regressions are adjusted for baseline level of the outcome. IntWMVs: internally based work-motive values. ExtWMVs: externally based work-motive values. f-IntWMV (fraction of total): IntWMV/total work-motive value score. **p < 0.01 and *p < 0.05, ns: non-significant.

IntWMV score was strongly associated with self-leadership both cross-sectionally and prospectively (Tables 3, 4).

*Effects of the relative contribution of internally based work-motive values*: We found that f-IntWMV (fraction of total motive value score) was strongly associated with reporting all work factors except fair leadership and social support (cross-sectional analyses) and role conflict (prospective analysis; Tables 3, 4). All associations were in a positive direction with the exception of that of role clarity.

### 3.3 Effects of the strength of externally based work-motive values (ExtWMVs) on the reporting of work characteristics

ExtWMV score did not influence reported levels of job demands or feedback from work. ExtWMV score was weakly associated with reported levels of fair leadership, but there was no prospective association (Tables 3, 4). Hence, our hypotheses pertaining to ExtWMVs were not supported by the results.

ExtWMV score was positively associated with reported levels of role clarity (both cross-sectionally and prospectively; Tables 3, 4) and weakly associated with both aspects of social support (cross-sectionally). ExtWMV score was negatively associated with decision control, role conflict, and self-leadership (only cross-sectional analyses).

### 3.4 Effects of the general strength of motive values (or a general tendency for reporting stronger responses): effects of total motive value score on the reporting of work characteristics

The total work-motive value score was positively associated with all work factors measured, except role conflict (Table 5). However, only role clarity, innovative climate, and self-leadership were prospectively influenced by the total motive-value score (Table 5).

**Table 5:** Associations between total work-motive value score and reported work characteristics.

Independent	Cross-sectional analyses		Prospective analyses	
	Estimate	95% CI	Estimate	95% CI
Control decisions	0.112	[0.085, 0.140]**	0.027	[−0.006, 0.060]ns
Empowering leadership	0.144	[0.106, 0.183]**	0.012	[−0.037, 0.060]ns
Innovative climate	0.207	[0.179, 0.234]**	0.048	[0.013, 0.084]**
Self-leadership	0.403	[0.375, 0.432]**	0.091	[0.055, 0.127]**
Quantitative demands	0.098	[0.070, 0.126]**	0.020	[−0.012, 0.053]ns
Learning demands	0.104	[0.079, 0.129]**	0.008	[−0.023, 0.039]ns
Feedback from work	0.149	[0.114, 0.184]**	0.019	[−0.026, 0.063]ns
Fair leadership	0.063	[0.042, 0.085]**	0.014	[−0.014, 0.043]ns
Social support from superior	0.146	[0.109, 0.183]**	0.015	[−0.031, 0.061]ns
Social support from co-workers	0.158	[0.128, 0.189]**	0.005	[−0.033, 0.043]ns
Role clarity	0.163	[0.135, 0.192]**	0.060	[0.026, 0.094]**
Role conflicts	0.000	[−0.030, 0.031]ns	0.011	[−0.026, 0.048]ns

**p < 0.01 and *p < 0.05, ns: non-significant.

## 4 Discussion

The present study found that managerial role, skill level, gender, and age are associated with internally based work-motive values (IntWMV, Table 2). These motive values in turn influenced the perception and appraisal of several specific work characteristics (Tables 3, 4). The total work-motive value score was cross-sectionally associated with all work factors except role conflicts, but only role clarity, innovative climate, and self-leadership showed significant prospective effects (Table 5).

Skill level and holding a managerial role were strongly associated with reporting a higher level of IntWMVs. Skill level corresponding to education/experience >13 years and being a top manager were also negatively associated with ExtWMV (Table 2). Effects on the total motive-value score were negligible. Motives and values pertaining to work may be bidirectionally associated with the level of education and acquiring managerial responsibilities. The very values and attitudes to develop one’s personality, use one’s imagination and creativity, and accomplish something worthwhile may motivate completing higher education and taking up managerial roles. On the other hand, higher levels of education and taking on leadership roles may possibly promote values related to personal development.

The present data showed that managers may differ from their subordinates in work-motive values and, consequently, differ in terms of goals and interests pertaining to work. This raises the question of whether leaders and managers face challenges in ascertaining and supporting the needs and interests of subordinates. Communicating and working with people with different work-motive values may require high levels of empathy and humility.

Female employees reported stronger total motive-value scores primarily due to relatively stronger ExtWMVs (Table 2). This finding may result from gender differences in values prioritizing family or gender differences in general attitudes toward safety and security. A third explanation is that females prioritize economic security and safety due to their perceptions of gender discrimination in the job market. It should be noted that we did not take seniority or weekly work hours into account, based on the assumption that these factors may be related to skill level.

Age was related to ExtWMVs and total motive-value scores (Table 2). However, the associations were weak, and there were no prospective associations.

The present study found that employees with internally based work-motive values (IntWMV, f-IntWMV) reported higher levels of several psychological work factors: control of decisions, empowering leadership, innovative climate, quantitative demands, feedback from work, and role conflicts, while there were negative associations with role clarity (Tables 3, 4). Prospective effects on learning demands, feedback, and role expectations were inconsistent. Therefore, motive values can influence survey measurements of several work characteristics of consequence for both research and practical assessments of risk. Motive values were weakly related to the perception of fair leadership and social interactions.

The hypothesis that internally based motive values (IntWMVs) are related to the perception of facilitators or barriers to personal development or accomplishing something worthwhile was confirmed by the finding that both IntWMV score and f-IntWMV (fraction of total motive value score) were associated with control of decisions, empowering leadership, and innovative climate. These consistent positive associations suggest that the employees with higher levels of IntWMVs are concerned with these particular work factors and/or that their values shape the appraisal of these factors. Alternatively, the finding that associations were positive may suggest that individuals in jobs with higher levels of these job characteristics also exhibit higher levels of IntWMVs, that is, that internally based motive values are a mediator between socioeconomic status and the appraisal and reporting of one’s work situation. However, we adjusted all analyses for skill level. Finally, reporting higher levels of IntWMVs and control, empowering leadership, and innovative climate may be a result of a third factor, for example, personality traits.

IntWMVs were also positively associated with quantitative demands and feedback from work suggesting that either IntWMVs are related to placing emphasis on input–outcome relationships, or that employees with higher IntWMVs tend to have jobs characterized by higher demands and more frequent feedback. These analyses were adjusted for skill level, but it is possible that internally based motive values mediate associations between socioeconomic status and appraisal of work characteristics as mentioned above.

Studies of the job characteristics model (JCM) reported that “growth need strength” moderated relationships between job characteristics and satisfaction in high-skilled workers (Hackman and Oldham, 1976; Loher et al., 1985). IntWMVs seem related to “growth need strength” of these studies, and the present study found that IntWMVs were positively associated with control and feedback, both of which are related to JCM factors.

IntWMVs and f-IntWMVs were strong predictors of self-leadership, that is, of employees’ autonomous behaviors (Stewart et al., 2011). ExtWMVs exhibited a weak statistically significant negative association. These associations were significant in both cross-sectional and prospective analyses taking baseline level of self-leadership into account, suggesting that IntWMVs is reflected in the employee’s behaviors or in his or her perceptions of own behaviors. It seems possible that IntWMVs and self-leadership are overlapping concepts or that IntWMV is a precondition for self-leadership to develop. A potential reverse effect was not ruled out.

ExtWMVs were positively associated with role clarity and showed weak–moderate positive associations with innovative climate, fair leadership, and social support, while the association with control of decisions was negative (Tables 3, 4). We hypothesized that ExtWMV, that is, instrumentally based motive values, is related to the appraisal of factors related to input–outcome relationships according to equity theory (Adams, 1963). We did not find significant associations with factors related to input (quantitative demands) or outcome (feedback, fair leadership, Table 4) and the proposed hypothesis did not receive support. An alternative hypothesis suggesting that employees reporting externally based motive values place less emphasis on work content in general, seems to receive support from these findings.

There are alternative general explanations for the present findings. Work-motive values may influence the perception and appraisal of work tasks, social interactions, and leader behaviors by four hypothetical general mechanisms. (I) Work-motive values, that is, terminal values, influence the interest, involvement, and commitment to a job and the significance the person assigns to certain value-relevant factors at work. Consequently, motive values may direct an employee’s *attention* to those aspects of the job that are relevant for satisfying motives (according to appraisal theory) and those work characteristics that are considered relevant to values are monitored closely. Having IntWMV may be associated with paying more attention to the contents of work tasks and work characteristics in order to appraise alignment with personal goals and interests (Tables 3, 4). On the other hand, ExtWMV may pertain to needs or priorities that are unrelated to the contents of work tasks, such as salary, security, and safety. Hence, job-task content may be of lower importance for employees with primarily externally based work-motive values. (II) IntWMV may be associated with a response style of *lower* tendency for midpoint responding, in turn increasing variance. Response styles may be defined as “tendencies to respond systematically to questionnaire items on some basis other than what the items were specifically designed to measure” (Baumgartner and Steenkamp, 2001) and may serve as a heuristic to minimize cognitive effort. Consequently, values may influence the effort put into evaluating one’s work situation and responding to surveys. Skill level and managerial role were associated with IntWMV, but there are conflicting findings on the relationships between the level of education and an extreme response style (Van Vaerenbergh and Thomas, 2013). Nevertheless, this explanation does not account for the direction of associations. (III) Work-motive values may influence self-selection into jobs that are congruent with one’s values and priorities. Finding positive associations between motive values and appraisals of work characteristics indicates that self-selection into the present job had successfully met motive values. (IV) Exhibiting IntWMVs may be associated with a positive attitude toward work in general. Consequently, these persons tend to report more positive appraisals of work characteristics.

### 4.1 Methodological considerations

The present study was based on validated measures of work characteristics (Dallner et al., 2000; Wännström et al., 2009). The work-characteristic questions and response alternatives were worded to avoid negative or positive connotations and influence of affect on responding (Askim and Knardahl, 2021). The study encompassed a rather large number of employees (cross-sectional analyses: *N* = 12,994; prospective analyses: *N* = 6,252). Response rates (defined as employees who provided response to all relevant factors as a percentage of all employees invited) were 48.1% in cross-sectional and 40.1% in prospective analyses. Respondents worked in a rather large number of organizations/businesses (cross-sectional analyses: 101; prospective analyses: 69) from both private and public sectors, with several types of jobs. Therefore, we are not aware of sources of selection bias. However, for evaluating external validity, one should consider the fact that the study was conducted in Norway, a country known for its strong emphasis on welfare, during a period of solid economy, and within a Scandinavian culture context.

The ExtWMV scale consisted of four items (“peaceful and orderly job,” “secure and provide regular income,” “safe and healthy physical work environment,” and “good salary and material goods”). One of these items, “good salary and material goods,” was not included in the “extrinsic motivation to work” factor of the QPS_Nordic_ (Dallner et al., 2000). There is a theoretical possibility that ExtWMV consists of two (or more) components: (i) safety/security and (ii) salary/remuneration/material goods. We did not pursue investigating these aspects.

Both motive values (independent variables) and work characteristics (dependent variables) were measured with surveys, that is, subjective reports. Since one objective of this study was to evaluate whether motive values influence the perception and appraisal and reporting of one’s work, subjective reports were essential for addressing the research questions. The present study did not attempt to assess an objective reality; hence, method bias due to subjective reporting (Podsakoff et al., 2012) should be of minor relevance. However, response styles such as acquiescence responding, extreme response style, or midpoint responding may produce response bias and common-method bias, thereby inflating associations. As discussed previously, we have not found evidence that gender or skill level should influence response styles (Van Vaerenbergh and Thomas, 2013). The affective state at the time of responding does not seem to influence subjective reports of these work factors to a significant degree (Askim and Knardahl, 2021). Separating the survey items that measure independent and dependent in space (different sections in the questionnaire) and time are recommendations for attenuating common-method bias (Podsakoff et al., 2012). Most of the associations for IntWMVs were also found with prospective analyses; hence, response biases if existing seem related to the individual’s values rather than to contextual or questionnaire issues.

### 4.2 Conclusion and implications

The present results show that individual motive values influence the appraisal and reporting of several aspects of psychosocial work characteristics. In particular, internally based work-motive values (IntWMVs) influence reports of control, empowering leadership, innovative climate, quantitative demands, and feedback from work, in addition to self-leadership. Therefore, measurements of work characteristics for research or for practical risk assessment should consider taking motive values into account.

Since gender, skill level, and managerial role influence motive values, it seems possible that motive values partially mediate associations between job roles and the experience of work characteristics. Managers may thus differ from their respective subordinates in work-motive values, and there may be differences in goals and interests. Leaders and managers may face the challenge of empathy in ascertaining and supporting the needs and interests of subordinates.

## Data availability statement

The data analyzed in this study is subject to the following licenses/restrictions: data are not publicly available due to the terms of participation. Analysis code and research materials may be made available upon request. Data were analyzed using R version 4.2.2 (R Core Team, 2022) and the package “lme4” (Bates et al., 2015). Requests to access these datasets should be directed to SK, stein.knardahl@stami.no.

## Ethics statement

The studies involving humans were approved by Norwegian Regional Committee for Medical and Health Research Ethics, Norwegian Data Inspectorate. The studies were conducted in accordance with the local legislation and institutional requirements. The participants provided their written informed consent to participate in this study. Written informed consent was obtained from the individual(s) for the publication of any potentially identifiable images or data included in this article.

## Author contributions

SK: Writing – original draft, Writing – review & editing, Project administration, Methodology, Funding acquisition, Conceptualization. JC: Writing – review & editing, Methodology, Formal analysis, Data curation.

## Appendix

**Table A1:** Descriptive statistics: means and standard deviations (range for all variables: 1 – 5).

Variable	Mean	SD
IntWMV	3.83	0.63
ExtWMV	3.77	0.58
Control decisions	2.98	0.78
Empowering leadership	3.13	1.02
Innovative climate	3.59	0.74
Self-leadership	3.29	0.75
Quantitative demands	2.94	0.76
Learning demands	2.54	0.64
Feedback from work	2.66	0.93
Fair leadership	3.13	0.53
Social support from superior	3.82	0.95
Social support from co-workers	4.12	0.79
Role clarity	4.19	0.76
Role conflicts	2.57	0.80

IntWMV, internally based work-motive values; ExtWMV, externally based work-motive values.
